# Two functional indel polymorphisms in the promoter region of the Brahma gene (*BRM*) and disease risk and progression-free survival in colorectal cancer

**DOI:** 10.1371/journal.pone.0198873

**Published:** 2018-06-12

**Authors:** Yajun Yu, Dangxiao Cheng, Patrick Parfrey, Geoffrey Liu, Sevtap Savas

**Affiliations:** 1 Discipline of Genetics, Faculty of Medicine, Memorial University, St. John’s, Newfoundland and Labrador, Canada; 2 Department of Medical Biophysics, University of Toronto, Toronto, Ontario, Canada; 3 Clinical Epidemiology Unit, Faculty of Medicine, Memorial University, St. John’s, Newfoundland and Labrador, Canada; 4 Division of Medical Oncology and Hematology, Department of Medicine, Princess Margaret Cancer Centre and University of Toronto, Toronto, Ontario, Canada; 5 Epidemiology, Dalla Lana School of Public Health, University of Toronto, Toronto, Ontario, Canada; 6 Discipline of Oncology, Faculty of Medicine, Memorial University, St. John’s, Newfoundland and Labrador, Canada; Sapporo Ika Daigaku, JAPAN

## Abstract

**Background and objective:**

The Brahma gene (*BRM*) encodes a catalytic ATPase subunit of the Switch/Sucrose non-fermentable (SWI/SNF) complex, which modulates gene expression and many important cellular processes. Two indel polymorphisms in the promoter region of *BRM* (*BRM-741* and *BRM-1321*) are associated with its reduced expression and the risk of susceptibility or survival outcomes in multiple solid cancers. In this study, we have examined these variants in relation to susceptibility and survival outcomes in colorectal cancer.

**Methods:**

Genotypes were obtained using TaqMan assays in 427 cases and 408 controls. Multivariate logistic and Cox regression models were fitted to examine the associations of the *BRM-741* and *BRM-1321* genotypes adjusting for relevant covariates. Sub-group analyses based on tumor location and patient sex were also performed. In all analyses, indels were examined individually as well as in combination.

**Results:**

Our results showed that there was no association between the *BRM* polymorphisms and the risk of colorectal cancer. However, genotype combinations of the *BRM-741* and *BRM-1321* variants were associated with the risk of colon cancer. Particularly, patients having at least one variant allele had increased risk of colon cancer when compared to patients with the double wild-type genotype. In the survival analyses, *BRM-741* heterozygosity was associated with longer progression-free survival time in the colorectal cancer patients. A stronger association was detected in the male patients under the recessive genetic model where the homozygosity for the variant allele of *BRM*-741 was associated with shorter progression-free survival time.

**Conclusions:**

Our analyses suggest that *BRM-741* and *BRM-1321* indels are associated with the risk of developing colon cancer and the *BRM-741* indel is associated with the disease progression in colorectal cancer patients, especially in the male patients. Although our results show a different relationship between these indels and colorectal cancer compared to other cancer sites, they also suggest that *BRM* and its promoter variants may have biological roles in susceptibility and survival outcomes in colorectal cancers. Performing further analyses in additional and larger cohorts are needed to confirm our conclusions.

## Introduction

Each year, around 1.4 million people are diagnosed with colorectal cancer and about 700,000 deaths occur because of it [1]. In Canada, around one in 15 people are expected to be diagnosed with this disease in their lifetime [2]. Worldwide, ~40–66% colorectal cancer patients do not survive 5-years after diagnosis [3]. Understanding factors, including genetic factors, that influence the susceptibility to this disease and patient prognosis can help improve its control and patient survival outcomes. For this reason, many studies have examined the associations of genetic variations with the risk of developing colorectal cancer or clinically important events after diagnosis [4–11].

*BRM* encodes Brahma, one of the two mutually exclusive DNA-dependent ATPase subunits of the SWI/SNF complex [12]. The SWI/SNF complex includes several subunits, exists in multiple forms with different subunit compositions, facilitates transcriptional regulation through remodeling of the chromatin, and is known to play critical roles in many important biological processes, such as cell proliferation and differentiation [13, 14]. Not surprisingly, several alterations of the multiple SWI/SNF complex subunits (including of *BRM*) have been identified in cancer, linking them to carcinogenesis or disease progression [14, 15].

Loss of BRM is often observed in various types of tumors [16–20], which is mainly mediated through epigenetic silencing [16]. Two promoter polymorphisms, *BRM*-741 and *BRM*-1321, are highly correlated with the expression levels of *BRM* [21]. Both of these polymorphisms are indel/repeating sequence variants [21]. *BRM*-741 consists of two (deletion or wild-type allele = Del) or three (insertion or variant allele = Ins) copies of a 7 bp long sequence (TATTTTT) located 741 bp upstream of the *BRM* transcription start site. *BRM*-1321, on the other hand, exists as either one (deletion or wild-type allele = Del) or two copies (insertion or variant allele = Ins) of a 6 bp long sequence (TTTTAA) located further upstream of the *BRM* transcription start site [21]. Variant sequences of these two polymorphisms are highly homologous to the binding site for myocyte enhancer factor-2 (MEF-2), which together with histone deacetylases (HDACs) has been shown to epigenetically silence the *BRM* gene [21, 22]. In examination of tissue samples, Liu et al. [21] associated the homozygosity for the variant allele (Ins/Ins) in either or both of the indels with the absence of BRM protein in both lung tumor and unaffected tissues. Examination by Gao et al. [23] showed that in both hepatocellular carcinoma tumors and non-tumor tissues the BRM expression levels decreased similarly with each Ins allele of *BRM*-1321. It is not known at the time being whether the Ins allele of *BRM*-741 has a similar effect on *BRM* expression as in the case of *BRM*-1321 (i.e. expression levels decreasing similarly with each copy of the Ins allele), but considering the fact that the *BRM* silencing is mediated through the binding of the MEF-2 and HDACs to the Ins alleles [21,22], it is a plausible possibility. Last but not least, both indels are linked to each other to varying degrees in different populations (D’ = 0.39–0.86) [20, 21, 23–26] and are common in Caucasians with similar minor allele frequencies (MAFs) of 45% [21].

Because *BRM*-741 and *BRM*-1321 can affect the expression of *BRM* and thus the activity of SWI/SNF, it is reasonable to suspect that these two polymorphisms may influence the risk or prognosis of human cancers. Supporting this, specific genotypes of either -741, -1321, or their combinations have been reported to be associated with the risk of lung [21, 27], head and neck [20, 27], and liver [23] cancers. Similar associations with the survival outcomes of lung [24], esophageal [25], hepatocellular [26] and pancreatic cancer [28] patients have also been detected. However, these two indels were not evaluated for their potential associations with the risk or survival outcomes in colorectal cancer before. In this study, we tested these associations in colorectal cancer cases and controls from Newfoundland.

## Methods

### Ethical approval

This study was approved by the Health Research Ethics Authority (HREA) of Newfoundland and Labrador (Reference numbers 09.106 and 15.294). Since this was a secondary use of data, no patient consent specific for this study was required.

### Study cohorts

Cases and controls recruited to Newfoundland Familial Colorectal Cancer Registry (NFCCR) were examined. NFCCR was described elsewhere in detail [29, 30]. In brief, participants (or their family members) provided consent to participate in NFCCR. A total of 750 stage 0-IV cases diagnosed between January 1999 and December 2003 were recruited. Age, sex and other related demographic information was collected at the time of recruitment. Access to medical records and blood or tissue samples were requested. Individuals free of colorectal cancer were enrolled as controls in the year of 2004 and 2005 by random-digit-dialing [31]. Controls were frequency-matched with the cases in terms of age and sex. In total, 720 controls were recruited. Blood samples and demographic information using questionnaires were collected at the time of recruitment. Cases who smoked cigarettes before the time of diagnosis were defined as ever-smokers while those did not smoke till this time point were defined as never-smokers. For controls, the time of recruitment was used as the time point to define ever-smoker and never-smoker status. Body mass index (BMI) was calculated based on the body mass and height information provided by the participants. For cases, these data were based on approximately one year before the diagnosis and for controls, these data were based on approximately two years before their recruitment.

Exclusion criteria for the study cohorts included: (1) cases and controls who were >75 years of age; (2) cases and controls who self-identified themselves as non-white or of mixed race; those who did not provide this information were also excluded; (3) cases who were diagnosed with stage 0 disease; (4) cases who were affected by Lynch syndrome, familial colorectal cancer type X (FCCX), or familial adenomatous polyposis (FAP); (5) cases who were the first, second, or third degree relatives with each other; in such a case one of the patients were randomly excluded. This information was based on a previously obtained genome-wide SNP genotype data [11] and was available for all but three patients; (6) controls who had a known first, second, or third degree relative in the case cohort; (7) controls who are known to have developed colorectal cancer after recruitment; (8) controls with no epidemiological/demographic data; and (9) cases or controls without genomic DNA extracted from blood samples. In the end, 427 cases and 408 controls passed these eligibility criteria. As for the survival analysis, one patient with no prognosis-related data was excluded. Characteristics of the cases and controls are summarized in Table 1.

**Table 1:** Distribution of baseline characteristics of the study cohorts.

Characteristics	Cases N (%)	Controls N (%)	* P value
**Total**	427 (100)	408 (100)	
^**†**^ **Age**			0.40
< 65 years	268 (62.76)	245 (60.05)	
≥ 65 years	158 (37.00)	163 (39.95)	
Unknown	1 (0.23)	-	
**Sex**			0.91
Female	172 (40.28)	166 (40.69)	
Male	255 (59.72)	242 (59.31)	
**Number of FDR with colorectal cancer**			**0.0004**
0	304 (71.19)	333 (81.62)	
At least 1	123 (28.81)	75 (18.38)	
**Smoking status**			0.09
Never	124 (29.04)	143 (35.05)	
Ever	296 (69.32)	265 (64.95)	
Unknown	7 (1.64)	-	
^**‡**^ **BMI**			0.35
Underweight and normal	119 (27.87)	127 (31.13)	
Overweight and obese	294 (68.85)	272 (66.67)	
Unknown	14 (3.28)	9 (2.21)	
^**§**^ **Disease stage**			-
I	76 (17.84)	-	
II	167 (39.20)	-	
III	140 (32.86)	-	
IV	43 (10.09)	-	
^**§**^ **Tumor location**			-
colon	280 (65.73)	-	
rectum	146 (34.27)	-	
^**§**^ **MSI status**			-
MSS\MSI-L	368 (86.38)	-	
MSI-H	40 (9.39)	-	
Unknown	18 (4.23)	-	
^**§**^ **Treatment with adjuvant chemotherapy**			-
No	189 (44.37)	-	
Yes	233 (54.69)	-	
Unknown	4 (0.94)	-	

BMI, body mass index; FDR, first-degree relative(s); MSI, microsatellite instability; MSI-H, microsatellite instability-high; MSI-L, microsatellite instability-low; MSS, microsatellite stable; N, number. P values < 0.05 are bolded.

* Two-sided χ^2^ test for comparison between cases and controls with available data.

^†^ Age is the age at diagnosis for cases, and age at recruitment for controls.

^‡^ Underweight, normal, overweight, and obese are defined as BMI <18.5, 18.5 ≤ BMI < 25, 25 ≤ BMI < 30, BMI ≥ 30, respectively. Categorization criterion was based on the information provided on the website of National Institutes of Health (https://www.nhlbi.nih.gov/health/educational/lose_wt/BMI/bmicalc.htm).

^§^ Total number of cases is 426.

### Follow-up

Patients were followed until the year 2010. The median follow up was 6.98 years (range: 2.00–10.88 years) with 95% confidence intervals (CIs) of 6.69–7.28 years (calculated based on reverse Kaplan-Meier method [32] using IBM SPSS Statistics-23). Data on vital status and occurrence of recurrence and metastasis were collected from various sources as explained in Negandhi et al. [33]. In brief, collection of prognostic data was performed by NFCCR. Clinical events of interest (i.e. recurrence/metastasis/death) were surveyed through and extracted/obtained from the patients’ medical records, the Newfoundland Cancer Treatment and Research Foundation database, or patient follow-up questionnaires.

### DNA genotyping

All NFCCR cases and controls with available DNA samples were genotyped for the *BRM* promoter indel polymorphisms (n = 493 for cases and n = 448 for controls). Genotyping for *BRM* -741 and *BRM* -1321 promoter region polymorphisms was performed by two custom designed Taqman assays (*BRM*-741: forward primer: 5’ TGGCAGGAACGTTCTTTGTG 3’; reverse primer: 5’ TGCCGGCTGAAACTTTTTCT 3’; probe for insertion: /56-FAM/TCCCTTTTCTA/ZEN/TTTTTTATTTTTTATTTTTTTACCTGGAA/3IABkFQ/; probe for wild-type: /5HEX/CCTCCCTTTTC/ZEN/TATTTTTTATTTTTTTACCTGGAAT/3IABkFQ/; *BRM*-1321: forward primer: 5’ CATACTTTTCATAACACTACTGCATAGGAACA 3’; reverse primer: 5’ TTTTATGAAGTGTGAAAGAATGTTAGGAGACT 3’; probe for insertion: /56-FAM/A+CT+CTTA+AAAT+T+AAAA+CTGT/3IABkFQ/; probe for wild-type: /5HEX/T+G+CTT+GA+CT+CTTAA+AAC/3IABkFQ/. TaqMan assay reaction condition for *BRM*-741 and -1321 polymorphisms was: 95°C 2 min followed by 40 cycles of 95°C for 6 sec / 60°C for 20 sec. Reaction volume for each sample was 5μl. PCR master mix was obtained from Kapabiosystems (Kapa probe fast qPCR kit, Cat#kk4702). For *BRM*-741 and *BRM*-1321, 9.58% and 19.26% of the DNA samples were genotyped twice and concordance rate was 100%. These methods had been previously compared with Sanger sequencing, and two other sets of primers and probes in 190 patients with 100% concordance.

A total of 831 (n = 424 of 427 cases, n = 407 of 408 controls) and 832 (n = 425 of 427 cases, n = 407 of 408 controls) individuals included into the study were successfully genotyped for the *BRM*-741 and *BRM*-1321 variants, respectively.

### Statistical analysis

Hardy-Weinberg equilibrium (HWE) calculations were performed using an online calculator (http://www.oege.org/software/hwe-mr-calc.shtml) [34]. D' and r^2^ for linkage disequilibrium (LD) between *BRM*-741 and *BRM*-1321 were calculated by using the LD function of genetics package [35] in R (ver3.2.4) [36]. Chi-squared test was used to examine the differences between cases and controls. All analyses were performed by using R (ver3.2.4) [36] unless otherwise specified.

Similar to other studies, deletions (Del) were assigned as wild-type alleles, and insertions (Ins) as variant alleles. Individual associations of the *BRM*-741 and *BRM*-1321 were analyzed under different genetic models (co-dominant, dominant, recessive and additive genetic models). Combination analyses involving both polymorphisms were performed as follows: **Category A)** the genotype categorizations used by the previous investigators [20, 21, 24, 25, 28]; **Category B)** double homozygous variant genotype (Ins/Ins+Ins/Ins) compared to others; **Category C)** double wild-type genotype (Del/Del+Del/Del) compared to others; and **Category D)** at least one homozygous variant genotype compared to others (shown in S1 Table; Categories A-D).

#### Association analyses

In the case-control study, unconditional logistic regression analyses were applied to test the associations between the indels and the risk of colorectal cancer. Known risk factors (age, sex, and number of first-degree relatives (FDR) with colorectal cancer) were included in multivariable models. Smoking status and BMI were sequentially examined using the log likelihood ratio test. We first examined smoking status and compared models with and without this variable. As the models were significantly different from each other (P values < 0.001) and the model with this variable had a smaller Akaike Information Criterion (AIC) value [37] (and, thus improved model's fit to data), smoking status was included as a covariate in the baseline model. We then examined BMI and obtained similar results. Thus, BMI was also included in the final baseline model as a covariate. Odds ratios (ORs) and 95% CIs for the genotypes were calculated under the multivariate logistic regression models adjusted for the baseline variables.

Cox Proportional Hazards (PH) regression method was used for survival analyses. The outcome of interest was progression-free survival that was defined as the time from diagnosis till the time of death, or, local or distant recurrence. Patients were censored if they experienced none of the events (death, recurrence or metastasis) till the last follow-up. The proportional hazard assumption was tested by using the cox.zph function [38] in R (ver3.2.4) [36]. Age was the only variable that violated the PH assumption (including genotypes), thus multivariate models were stratified by age. Other model covariates included disease stage, tumor location, microsatellite instability (MSI) status, and treatment status (adjuvant chemotherapy Yes/No). Their independent associations with the outcome were confirmed in a multivariable baseline model. Hazard ratios (HRs) and corresponding 95% CIs for the genotype categories were estimated under the age-stratified multivariate Cox models adjusting for these baseline variables.

#### Sub-cohort analyses

To explore whether the associations of these indels vary by sex and tumor location (colon, rectum), we also performed sub-cohort analyses separately (S2–S5 Tables). Adjustments in sub-cohort analyses were done by the covariates previously selected, except for the covariate sex in the risk analyses in male and female sub-cohorts, and tumor location in survival analyses in colon and rectal cancer sub-cohorts. In addition, MSI was not included as a covariate in survival analyses of rectal cancer cases because there were only two rectal cancer patients with microsatellite instability-high (MSI-H) tumors.

## Results

### Minor allele frequencies, Hardy-Weinberg equilibrium test, and linkage disequilibrium between the *BRM*-741 and *BRM*-1321 indels

Minor allele frequencies of *BRM*-741 and *BRM*-1321 were 48% and 44% in controls and 47% and 43% in cases, respectively. Both *BRM*-741 and *BRM*-1321 genotype frequencies satisfied the HWE in controls. D' and the r^2^ between the *BRM*-741 and *BRM*-1321 were lower than 0.8 in cases (D' = 0.48; r^2^ = 0.20) and controls (D' = 0.58; r^2^ = 0.29), indicating the two polymorphisms were not highly correlated with each other in this population.

### Associations of the *BRM*-741 and *BRM*-1321 indels with the susceptibility to colorectal cancer

#### Case-control analyses in colorectal cases and controls

Cases (n = 427) and controls (n = 408) were comparable to each other in terms of frequency distribution of age, sex, smoking status, and BMI, except the number of FDR affected by colorectal cancer as expected (Table 1). After adjusting for age, sex, number of FDR, smoking status, and BMI, *BRM* indels were not associated with the risk of colorectal cancer when analyzed alone or together (Table 2).

**Table 2:** *BRM* promoter indels and colorectal cancer risk.

Variable	Genotypes	Cases N (%)	Controls N (%)	OR (95% CI)	* P value
***BRM-741***					
	**Co-dominant model**				
	Del/Del (wild-type)	119 (27.87)	113 (27.70)	1 (reference)	
	Ins/Del	215 (50.35)	201 (49.26)	1.09 (0.78, 1.52)	0.61
	Ins/Ins	90 (21.08)	93 (22.79)	0.96 (0.64, 1.44)	0.85
	Unknown	3 (0.70)	1 (0.25)		
	**Dominant model**				
	Del/Del	119 (27.87)	113 (27.70)	1 (reference)	
	Ins/Ins + Ins/Del	305 (71.43)	294 (72.06)	1.05 (0.77, 1.44)	0.76
	Unknown	3 (0.70)	1 (0.25)		
	**Recessive model**				
	Ins/Del + Del/Del	334 (78.22)	314 (76.96)	1 (reference)	
	Ins/Ins	90 (21.08)	93 (22.79)	0.91 (0.65, 1.28)	0.59
	Unknown	3 (0.70)	1 (0.25)		
	^**†**^ **Additive model**				
	Del/Del	119 (27.87)	113 (27.70)	0.99 (0.81, 1.21)	0.90
	Ins/Del	215 (50.35)	201 (49.26)		
	Ins/Ins	90 (21.08)	93 (22.79)		
	Unknown	3 (0.70)	1 (0.25)		
***BRM-1321***					
	**Co-dominant model**				
	Del/Del (wild-type)	136 (31.85)	135 (33.09)	1 (reference)	
	Ins/Del	213 (49.88)	188 (46.08)	1.20 (0.87, 1.65)	0.27
	Ins/Ins	76 (17.80)	84 (20.59)	0.93 (0.62, 1.39)	0.73
	Unknown	2 (0.47)	1 (0.25)		
	**Dominant model**				
	Del/Del	136 (31.85)	135 (33.09)	1 (reference)	
	Ins/Ins + Ins/Del	289 (67.68)	272 (66.67)	1.11 (0.83, 1.51)	0.48
	Unknown	2 (0.47)	1 (0.25)		
	**Recessive model**				
	Ins/Del + Del/Del	349 (81.73)	323 (79.17)	1 (reference)	
	Ins/Ins	76 (17.80)	84 (20.59)	0.84 (0.58, 1.19)	0.32
	Unknown	2 (0.47)	1 (0.25)		
	^**†**^ **Additive model**				
	Del/Del	136 (31.85)	135 (33.09)	0.99 (0.81, 1.21)	0.93
	Ins/Del	213 (49.88)	188 (46.08)		
	Ins/Ins	76 (17.80)	84 (20.59)		
	Unknown	2 (0.47)	1 (0.25)		
^**‡**^ ***BRM-741* and *BRM-1321*** **genotype combinations**					
	**Category A.**				
	Double wild-type genotype	73 (17.10)	81 (19.85)	1 (reference)	
	No homozygous variant genotype	223 (52.22)	196 (48.04)	1.36 (0.92, 2.00)	0.12
	One homozygous variant genotype	91 (21.31)	81 (19.85)	1.32 (0.84, 2.08)	0.23
	Double homozygous variant genotype	37 (8.67)	48 (11.76)	0.90 (0.51, 1.56)	0.70
	Unknown	3 (0.70)	2 (0.49)		
	**Category B.**				
	Other genotype combinations	387 (90.63)	358 (87.75)	1 (reference)	
	Double homozygous variant genotype	37 (8.67)	48 (11.76)	0.71 (0.44, 1.13)	0.15
	Unknown	3 (0.70)	2 (0.49)		
	**Category C.**				
	Double wild-type genotype	73 (17.10)	81 (19.85)	1 (reference)	
	Other genotype combinations	351 (82.20)	325 (79.66)	1.28 (0.89, 1.84)	0.19
	Unknown	3 (0.70)	2 (0.49)		
	**Category D.**				
	Other genotype combinations	296 (69.32)	277 (67.89)	1 (reference)	
	At least one homozygous variant genotype	128 (29.98)	129 (31.62)	0.93 (0.68, 1.26)	0.63
	Unknown	3 (0.70)	2 (0.49)		

CI, confidence interval; Del, deletion; Ins, insertion; N, number; OR, odds ratio.

* Adjusted for age, sex, number of first degree relatives with colorectal cancer, smoking status, and body mass index. Please note that final models include only the patients with the available covariate data. For further information on genotype combinations/categories, please refer to Methods/S1 Table.

^†^ Ins/Ins vs Ins/Del vs Del/Del.

^‡^ Homozygous variant genotype is Ins/Ins genotype.

#### Case-control analyses in the sub-cohorts

In multivariate analyses, significant associations were found only in the colon cases and when the *BRM*-741 and *BRM*-1321 indel genotypes were analyzed together (Table 3). Specifically, compared to double wild-type genotype (Del/Del+Del/Del), no homozygous or one homozygous variant genotypes were associated with the increased risk of colon cancer (no homozygous variant genotype; OR [95% CI] = 1.65 [1.05–2.63]; P value = 0.03; one homozygous variant genotype; OR [95% CI] = 1.77 [1.05–3.01]; P value = 0.03; Category A). Additionally, compared to the double wild-type genotype (Del/Del+Del/Del), combined genotypes that included at least one variant allele were associated with increased risk of colon cancer (OR [95% CI] = 1.60 [1.04–2.50]; P value = 0.03; Category C). There were no associations detected in the rectal cancer, female, or male cancer sub-cohorts (S2 and S3 Tables).

**Table 3:** Associations between *BRM* promoter indels and colon cancer risk.

Sub-cohort	Variable	Genotypes	Cases N (%)	Controls N (%)	OR (95% CI)	* P value
**Colon cases + Controls**	^**†**^ ***BRM-741* and *BRM-1321* genotype combination**					
		**Category A.**				
		Double wild-type genotype	41 (14.64)	81 (19.85)	1 (reference)	
		No homozygous variant genotype	148 (52.86)	196 (48.04)	1.65 (1.05, 2.63)	**0.03**
		One homozygous variant genotype	64 (22.86)	81 (19.85)	1.77 (1.05, 3.01)	**0.03**
		Double homozygous variant genotype	25 (8.93)	48 (11.76)	1.14 (0.60, 2.15)	0.69
		Unknown	2 (0.71)	2 (0.49)		
		**Category C.**				
		Double wild-type genotype	41 (14.64)	81 (19.85)	1 (reference)	
		Other genotype combinations	237 (84.64)	325 (79.66)	1.60 (1.04, 2.50)	**0.03**
		Unknown	2 (0.71)	2 (0.49)		

CI, confidence interval; N, number; OR, odds ratio. P values < 0.05 are bolded.

* Adjusted for age, sex, number of first degree relatives with colorectal cancer, smoking status and body mass index. Please note that final models include only the patients with the available covariate data. For further information on genotype combinations/categories, please refer to Methods/S1 Table.

^†^ Homozygous variant genotype is Ins/Ins genotype.

Only the results with P value less than 0.05 are shown in this table; all results obtained in the sub-cohort analyses are shown in S2 and S3 Tables.

### Associations of the *BRM*-741 and *BRM*-1321 indels with progression-free survival in colorectal cancer

#### Survival analyses in the colorectal cancer cases

**Fig 1:**
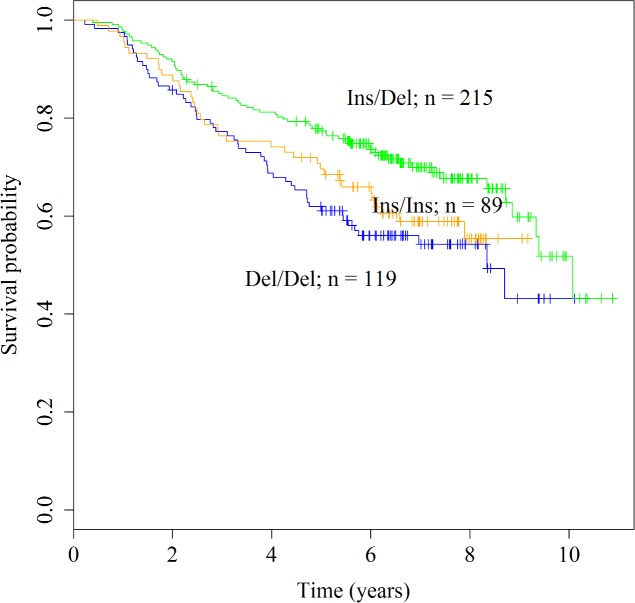
Kaplan-Meier curves for the *BRM*-741 indel under the co-dominant genetic model in the colorectal cancer cases. P value of the log-rank test is 0.017.

Results are summarized in Table 4. The only association was detected under the co-dominant genetic model where the heterozygosity for the *BRM*-741 indel was significantly associated with longer progression-free survival time when compared to wild-type genotype (Ins/Del vs Del/Del; HR [95% CI] = 0.67 [0.45, 0.98]; P value = 0.04; Fig 1). This association was independent of age, disease stage, tumor location, MSI and adjuvant chemotherapy status.

**Table 4:** *BRM* promoter indels and progression-free survival in colorectal cancer.

Variable	Genotypes	Cases N (%)	P value for PH assumption test	HR (95% CI)	* P value
***BRM-741***					
	**Co-dominant model**				
	Del/Del (wild-type)	119 (27.93)		1 (reference)	
	Ins/Del	215 (50.47)	0.72	0.67 (0.45, 0.98)	**0.04**
	Ins/Ins	89 (20.89)	0.95	0.97 (0.62, 1.51)	0.89
	Unknown	3 (0.70)			
	**Dominant model**				
	Del/Del	119 (27.93)		1 (reference)	
	Ins/Ins + Ins/Del	304 (71.36)	0.86	0.75 (0.53, 1.07)	0.12
	Unknown	3 (0.70)			
	**Recessive model**				
	Ins/Del + Del/Del	334 (78.40)		1 (reference)	
	Ins/Ins	89 (20.89)	0.81	1.24 (0.85, 1.82)	0.27
	Unknown	3 (0.70)			
	^**†**^ **Additive model**				
	Del/Del	119 (27.93)			
	Ins/Del	215 (50.47)	0.96	0.96 (0.75, 1.21)	0.72
	Ins/Ins	89 (20.89)			
	Unknown	3 (0.70)			
***BRM-1321***					
	**Co-dominant model**				
	Del/Del (wild-type)	136 (31.92)		1 (reference)	
	Ins/Del	212 (49.77)	0.45	0.95 (0.66, 1.36)	0.76
	Ins/Ins	76 (17.84)	0.64	0.98 (0.61, 1.58)	0.93
	Unknown	2 (0.47)			
	**Dominant model**				
	Del/Del	136 (31.92)		1 (reference)	
	Ins/Ins + Ins/Del	288 (67.61)	0.44	0.95 (0.68, 1.34)	0.79
	Unknown	2 (0.47)			
	**Recessive model**				
	Ins/Del + Del/Del	348 (81.69)		1 (reference)	
	Ins/Ins	76 (17.84)	0.88	1.01 (0.66, 1.55)	0.96
	Unknown	2 (0.47)			
	^**†**^ **Additive model**				
	Del/Del	136 (31.92)			
	Ins/Del	212 (49.77)	0.54	0.98 (0.78, 1.24)	0.88
	Ins/Ins	76 (17.84)			
	Unknown	2 (0.47)			
^**‡**^ ***BRM-741* and *BRM-1321*** **genotype combinations**					
	**Category A.**				
	Double wild-type genotype	73 (17.14)		1 (reference)	
	No homozygous variant genotype	223 (52.35)	0.56	0.66 (0.43, 1.00)	0.05
	One homozygous variant genotype	90 (21.13)	0.60	0.72 (0.44, 1.18)	0.19
	Double homozygous variant genotype	37 (8.69)	0.58	1.02 (0.55, 1.89)	0.96
	Unknown	3 (0.70)			
	**Category B.**				
	Other genotype combinations	386 (90.61)		1 (reference)	
	Double homozygous variant genotype	37 (8.69)	0.60	1.39 (0.81, 2.38)	0.24
	Unknown	3 (0.70)			
	**Category C.**				
	Double wild-type genotype	73 (17.14)		1 (reference)	
	Other genotype combinations	350 (82.16)	0.79	0.71 (0.48, 1.05)	0.09
	Unknown	3 (0.70)			
	**Category D.**				
	Other genotype combinations	296 (69.48)		1 (reference)	
	At least one homozygous variant genotype	127 (29.81)	0.51	1.07 (0.76, 1.52)	0.69
	Unknown	3 (0.70)			

CI, confidence interval; Del, deletion; HR, hazard ratio; Ins, insertion; N, number; PH, proportional hazard. P values < 0.05 are bolded. For further information on genotype combinations/categories, please refer to Methods/S1 Table.

* Results by age stratified Cox models adjusted for disease stage, tumor location, microsatellite instability (MSI) status, and treatment status (adjuvant chemotherapy Yes or No).

^†^ Ins/Ins vs Ins/Del vs Del/Del.

^‡^ Homozygous variant genotype is Ins/Ins genotype.

#### Survival analyses in the sub-cohorts

In the male colorectal cancer cohort, *BRM*-741 indel was associated with the progression-free survival time under the co-dominant and recessive genetic models (S4 Table). Similar to the results obtained in the entire patient cohort (Table 4), under the co-dominant genetic model heterozygosity for *BRM*-741 was associated with longer progression-free survival time compared to wild-type genotype (HR [95% CI] = 0.54 [0.34, 0.88]; P value = 0.01). This pattern was also evident in the Kaplan-Meier curves where the male patients with the wild-type Del/Del and the homozygous Ins/Ins genotypes had similar survival probabilities compared to heterozygous Ins/Del individuals who had better survival probability (S1 Fig). A stronger association was detected under the recessive genetic model, where the homozygosity for the *BRM*-741 Ins allele was associated with decreased progression-free survival time compared to other genotypes (HR [95% CI] = 1.84 [1.17, 2.90]; P value = 0.009; S2 Fig). These associations were restricted to the male patients and were not detected in female, colon, or rectal cancer cases (S4 and S5 Tables).

## Discussion

In this study, for the first time we have investigated whether two functional variants (*BRM*-741 and *BRM*-1321) located in the promoter region of the *BRM* gene were associated with the susceptibility to develop colorectal cancer and survival times of the patients. Our results show that presence of at least one variant allele in both of these indels are associated with the increased risk of colon but not rectal cancer. Our results also show that *BRM*-741 may be associated with progression-free survival time in colorectal cancer patients, particularly in the male patients.

*BRM* codes for one of the two ATPase subunits of the SWI/SNF complex [12]. Two indel polymorphisms in the promoter region of *BRM*, *BRM*-741 and *BRM*-1321, have been shown to be associated with down-regulation of this gene [21], and therefore may affect the function of the SWI/SNF complex and cellular processes regulated by it. Some of these cellular processes are related to cancer, such as cell proliferation and differentiation [39], making these two genetic variants functionally interesting in cancer research. These variants are not included in many of the genotyping platforms and are not in high LD with other platform polymorphisms to be accurately imputed [21]. This means that the potential associations of these two *BRM* variants may have been missed in genome-wide studies, including one of ours in the NFCCR patient cohort [11]. A number of research groups genotyped and studied the associations of *BRM*-741 and *BRM*-1321 indels with the risk or survival outcomes in various solid cancers. As it is summarized below, while the particular genotypes that are associated with the risk of disease or clinical outcomes are not consistent across different cancer sites, it has been so far consistent that when an association is detected, the variant allele containing genotypes were associated with increased risk of disease/clinical events compared to the homozygous wild-type genotypes. These findings suggest that down-regulation of *BRM* may have a role in carcinogenesis or progression in these cancers.

Studies published so far have showed that either or both of the *BRM*-741 and *BRM*-1321 indels are associated with the risk of development of cancer in multiple, but not all, tissues examined. For example, in stage I-II lung and head and neck cancer patients, one study identified the association of the double homozygous variant genotype with increased disease risk [27]. Two other studies involving stage I-IV patients reported similar associations, in addition to associations of -741 (Ins/Ins genotype) and -1321 (Ins allele containing genotypes), with increased risks of lung cancer [21] as well as head and neck cancers [20]. Additionally, in two separate patient cohorts from Asia, *BRM*-741 was not found to be associated with the disease risk, whereas both the heterozygosity and homozygosity for the *BRM*-1321 Ins allele were associated with increased risk of liver cancer [23]. However, these associations were not detected in a Canadian cohort in a recent study [26]. Additionally, no associations were found between the two indels and the disease risk in pancreatic cancer (when analyzed either alone or in combination) [28], or in early stage esophageal cancer patients (when analyzed in combination) [27].

In colorectal cancer patients, including the male and female sub-cohorts, our multivariate analyses detected no associations of the *BRM*-741 and *BRM*-1321 indels with the disease risk when these variants were analyzed individually or in combination. However, when the analysis was restricted to the colon cancer patients, genotypes containing at least one variant allele were associated with increased risk of colon cancer compared to the double wild-type genotype (Del/Del+Del/Del) (Table 3 –Category C). These associations were independent of age, sex, number of first degree relatives with colorectal cancer, smoking status, and body-mass index. Additionally, as also shown in Table 3 (Category A), no homozygous and one homozygous genotypes were associated with increased risk of colon cancer compared to double wild type genotype. While we have not observed the association of the double homozygous variant genotype with increased cancer risk compared to double homozygous wild type genotype (likely because of the rarity of this genotype in our cases and controls; Table 3 –Category A), the fact that the presence of the variant alleles associates with increased colon cancer risk is biologically in line with the findings in other cancers. Therefore, similar to other cancer sites (e.g. lung and head and neck cancers [20, 21, 27]) our results suggest that the loss or reduced expression of *BRM* may increase the colon cancer risk. Interestingly, another gene coding for a subunit of the SWI/SNF complex (*ARID1A*/*BAF250A*) has been found to have frameshift or nonsense mutations in up to 10% of colon tumors [40], suggesting that abnormalities in ARID1A protein may have a role colon carcinogenesis. Together with our results, these findings suggest the possible involvement of the SWI/SNF complex in colon carcinogenesis. Overall, once confirmed in other patient cohorts our results may have significant implications for understanding the biological functions of the *BRM* gene and the SWI/SNF complex, and their potential roles in pathogenesis or treatment of colon cancer. In contrast, there was no evidence of associations of the *BRM* indels with the risk of rectal cancer. This may be attributed to insufficient power in the rectal cancer cohort (n = 146), or the fact that colon and rectal cancers are separate cancer sites arising in distinct tissues characterized with different pathogenesis and molecular alterations [41]. Further cohort and/or molecular studies can be valuable in addressing this hypothesis.

Similar to susceptibility studies, associations of the *BRM*-741 and *BRM*-1321 indels with survival outcomes have been reported in multiple cancer sites. For example, in pancreatic as well as in esophageal cancers, one or two copies of the indel variant alleles (Ins/Del, Ins/Ins) or double homozygous variant genotype (Ins/Ins+Ins/Ins) were associated with reduced overall survival time [25, 28]. Additionally, in two separate stage III-IV non-small cell lung cancer cohorts, homozygosity for the variant alleles of either indels as well as the double homozygous variant genotype were associated with shortened overall and progression-free survival time [24]. A recent study on liver cancer patients also showed similar associations between overall survival time and these indel variants [26]. In our study, no associations were detected between the progression-free survival time of the patients and the *BRM*-1321 genotypes or the genotype combinations of the *BRM*-741 and *BRM*-1321 indels. However, associations were detected for the *BRM*-741 genotypes. Specifically, when compared to the wild-type genotype (Del/Del), heterozygosity for the *BRM*-741 indel was associated with longer progression-free survival time in the colorectal cancer cohort independent of age, disease stage, tumor location, MSI and adjuvant treatment status (Table 4; Fig 1). A similar association was also detected in the male colorectal cancer patients (S4 Table; S1 Fig). Based on the previous studies on other cancers, we would expect the wild-type genotype to have better survival outcomes compared to the genotypes that include the variant allele. However, our results do not support this assumption. We also note that it is possible that the small sample size in the wild-type homozygous genotype group may have led to missing a possible association. In addition, under the recessive genetic model we found that the male patients who had the Ins/Ins genotype of *BRM*-741 had shorter survival times compared to the rest of the male patients (S4 Table; S2 Fig). This association was not detected in the female patients (p > 0.05: S4 Table). However, as shown in S3 Fig, while it did not reach significance, an opposite effect of the Ins/Ins allele in the female patients was observable, suggesting that the prognostic associations of the *BRM*-741 may be different between male and female colorectal cancer patients. This opens new research avenues for future studies that can help dissect the biological basis of sex-based differences in colorectal cancer outcomes.

Strengths/limitations of this study can be summarized as follows: replications in independent patient cohorts are required to rule out false-positive associations and to confirm our results; death from any cause was used as one of the endpoints as the cause of death information was not available for all patients; and the low frequency of the double homozygous variant genotype has possibly prevented examination/detection of its potential associations in our cohort, thus analysis of larger patient cohorts are needed. However, to our knowledge, this is the first study that investigated the association between the *BRM*-741 and *BRM*-1321 promoter variations and disease risk and patient survival outcomes in colorectal cancer; the patient cohort is a well described cohort with long follow-up time (median: 6.98 years); a comprehensive investigation has been conducted including application of multiple genetic models and sub-group analyses; and more importantly, in the survival analysis the proportional hazard assumption of the Cox regression method has been assessed and appropriate models have been constructed, which makes our estimations more reliable [42, 43].

## Conclusions

In conclusion, our results suggest the potential involvement of *BRM* in colon cancer pathogenesis and colorectal cancer progression. Analyses in larger and additional patient cohorts are needed to verify our results.

## Supporting information

S1 Table Definition of genotype combination categories. (PDF)

S2 Table Results of the multivariate logistic regression analyses for colon and rectal cancer patients. (PDF)

S3 Table Results of the multivariate logistic regression analyses for male and female patients. (PDF)

S4 Table Results of the age-stratified multivariate Cox regression (survival) analyses in the male and female sub-cohorts. (PDF)

S5 Table Results of the age-stratified multivariate Cox regression (survival) analyses in the colon and rectal cancer sub-cohorts. (PDF)

S1 Fig Kaplan-Meier curves for the *BRM*-741 indel under the co-dominant genetic model in the male colorectal cancer cases. (TIFF)

S2 Fig Kaplan-Meier curves for the *BRM*-741 indel under the recessive genetic model in the male colorectal cancer cases. (TIFF)

S3 Fig Kaplan-Meier curves for the *BRM-*741 indel under the recessive genetic model in the female colorectal cancer cases. (TIFF)
