# Supplementary material for: Two functional indel polymorphisms in the promoter region of the Brahma gene (BRM) and disease risk and progression-free survival in colorectal cancer
Source: PLoS One. 2018 Jun 12;13(6):e0198873. doi: 10.1371/journal.pone.0198873 (PMC5997361; doi:10.1371/journal.pone.0198873)
Supplement: S1 Table — (PDF) [file pone.0198873.s001.pdf]

S1 Table. Definition of genotype combination categories.

Categories	Genotype combination [*BRM*-741 + *BRM*-1321]	References
**Category A.**		
Double wild-type genotype	[Del/Del + Del/Del]	1-5
No homozygous variant genotype	[Ins/Del + Del/Del] or<br>[Del/Del + Ins/Del] or<br>[Ins/Del + Ins/Del]	
One homozygous variant genotype	[Ins/Ins + Del/Del] or<br>[Ins/Ins + Ins/Del] or<br>[Del/Del + Ins/Ins] or<br>[Ins/Del + Ins/Ins]	
Double homozygous variant genotype	[Ins/Ins + Ins/Ins]	
**Category B.**		
Double homozygous variant genotype	[Ins/Ins + Ins/Ins]	
Others	[Del/Del + Del/Del] or<br>[Ins/Del + Del/Del] or<br>[Del/Del + Ins/Del] or<br>[Ins/Del + Ins/Del] or<br>[Ins/Ins + Del/Del] or<br>[Ins/Ins + Ins/Del] or<br>[Del/Del + Ins/Ins] or<br>[Ins/Del + Ins/Ins]	
**Category C.**		
Double wild-type genotype	[Del/Del + Del/Del]	
Others	[Ins/Del + Del/Del] or<br>[Del/Del + Ins/Del] or<br>[Ins/Del + Ins/Del] or<br>[Ins/Ins + Del/Del] or<br>[Ins/Ins + Ins/Del] or	

	[Del/Del + Ins/Ins] or<br>[Ins/Del + Ins/Ins] or<br>[Ins/Ins + Ins/Ins]
**Category D.**	
At least one homozygous variant genotype	[Ins/Ins + Del/Del] or<br>[Ins/Ins + Ins/Del] or<br>[Del/Del + Ins/Ins] or<br>[Ins/Del + Ins/Ins] or<br>[Ins/Ins + Ins/Ins]
Others	[Del/Del + Del/Del] or<br>[Ins/Del + Del/Del] or<br>[Del/Del + Ins/Del] or<br>[Ins/Del + Ins/Del]

References

1. Korpanty GJ, Eng L, Qiu X, Faluyi OO, Renouf DJ, Cheng D, Patel D, Chen Z, Tse BC, Knox JJ, Dodbiba L, Teichman J, Azad AK, Wong R, Darling G, Reisman D, Cuffe S, Liu G, Xu W. Association of *BRM* promoter polymorphisms and esophageal adenocarcinoma outcome. *Oncotarget* 2017; 8(17):28093–28100.
2. Liu G, Cuffe S, Liang S, Azad AK, Cheng L, Brhane Y, Qiu X, Cescon DW, Bruce J, Chen Z, Cheng D, Patel D, Tse BC, Laurie SA, Goss G, Leighl NB, Hung R, Bradbury PA, Seymour L, Shepherd FA, Tsao MS, Chen BE, Xu W, Reisman DN. *BRM* promoter polymorphisms and survival of advanced non–small cell lung cancer patients in the Princess Margaret cohort and CCTG BR. 24 trial. *Clin Cancer Res* 2017; 23(10):2460-2470.
3. Liu G, Gramling S, Munoz D, Cheng D, Azad AK, Mirshams M, Chen Z, Xu W, Roberts H, Shepherd FA, Tsao MS, Reisman D. Two novel *BRM* insertion promoter sequence variants are associated with loss of *BRM* expression and lung cancer risk. *Oncogene* 2011; 30(29):3295-3304.

4. Segedi M, Anderson LN, Espin-Garcia O, Borgida A, Bianco T, Cheng D, Chen Z, Patel D, Brown MC, Xu W, Reisman D, Gallinger S, Cotterchio M, Hung R, Liu G, Cleary SP. *BRM* polymorphisms, pancreatic cancer risk and survival. *Int J Cancer* 2016; 139(11):2474-2481.
5. Wang JR, Gramling SJ, Goldstein DP, Cheng D, Chen D, Azad AK, Tse A, Hon H, Chen Z, Mirshams M, Simpson C, Huang SH, Marquez S, O'Sullivan B, Liu FF, Roberts H, Xu W, Brown DH, Gilbert RW, Gullane PJ, Irish JC, Reisman DN, Liu G. Association of two *BRM* promoter polymorphisms with head and neck squamous cell carcinoma risk. Carcinogenesis 34(5):1012-1017.
