# Supplementary material for: Two functional indel polymorphisms in the promoter region of the Brahma gene (BRM) and disease risk and progression-free survival in colorectal cancer
Source: PLoS One. 2018 Jun 12;13(6):e0198873. doi: 10.1371/journal.pone.0198873 (PMC5997361; doi:10.1371/journal.pone.0198873)
Supplement: S2 Table — (PDF) [file pone.0198873.s002.pdf]

S2 Table. Results of the multivariate logistic regression analyses for colon and rectal cancer patients.

**A. Colon cases (n=280) + controls (n=408)**					
			**95% CI**		
**Variables**	**Category**	***OR**	**lower**	**higher**	**p value**
BRM-741 (co-dominant model; 0=Del/Del; 1=Ins/Del; 2=Ins/Ins)	1 vs 0	1.39	0.95	2.05	0.10
	2 vs 0	1.23	0.78	1.96	0.38
BRM-1321 (co-dominant model; 0=Del/Del; 1=Ins/Del; 2=Ins/Ins)	1 vs 0	1.35	0.94	1.95	0.11
	2 vs 0	1.12	0.71	1.77	0.61
Genotype combination of *BRM*-741 and *BRM*-1321 (0=Both Del/Del; 1=No Ins/Ins; 2=One Ins/Ins; 3=Both Ins/Ins)	1 vs 0	1.65	1.05	2.63	**0.03**
	2 vs 0	1.77	1.05	3.01	**0.03**
	3 vs 0	1.14	0.60	2.15	0.69
Genotype combination of *BRM*-741 and *BRM*-1321 (0=Others; 1=Both Ins/Ins)	1 vs 0	0.74	0.43	1.25	0.27
Genotype combination of *BRM*-741 and *BRM*-1321 (0=Both Del/Del; 1=Others)	1 vs 0	1.60	1.04	2.51	**0.03**
Genotype combination of *BRM*-741 and *BRM*-1321 (0=Others; 1=At least one Ins/Ins)	1 vs 0	1.05	0.75	1.47	0.78
BRM-741 (dominant model; 0=Del/Del; 1=Others)	1 vs 0	1.34	0.93	1.94	0.12
BRM-741 (recessive model; 0=Others; 1=Ins/Ins)	1 vs 0	0.99	0.67	1.44	0.95
BRM-741 (additive model; 0=Del/Del; 1=Ins/Del; 2=Ins/Ins)	2 vs 1 vs 0	1.12	0.89	1.40	0.35
BRM-1321 (dominant model; 0=Del/Del; 1=Others)	1 vs 0	1.28	0.91	1.81	0.16
BRM-1321 (recessive model; 0=Others; 1=Ins/Ins)	1 vs 0	0.94	0.63	1.38	0.74
BRM-1321 (additive model; 0=Del/Del; 1=Ins/Del; 2=Ins/Ins)	2 vs 1 vs 0	1.09	0.87	1.36	0.47
**B. Rectum cases (n=146) + controls (n=408)**					
			**95% CI**		
**Variables**	**Category**	***OR**	**lower**	**higher**	**p value**
BRM-741 (co-dominant model; 0=Del/Del; 1=Ins/Del; 2=Ins/Ins)	1 vs 0	0.73	0.47	1.14	0.16
	2 vs 0	0.62	0.35	1.08	0.09
BRM-1321 (co-dominant model; 0=Del/Del; 1=Ins/Del; 2=Ins/Ins)	1 vs 0	0.95	0.62	1.48	0.83
	2 vs 0	0.62	0.34	1.11	0.11

Genotype combination of *BRM*-741 and *BRM*-1321<br>(0=Both Del/Del; 1=No Ins/Ins; 2=One Ins/Ins; 3=Both Ins/Ins)	1 vs 0<br>2 vs 0<br>3 vs 0	0.99<br>0.72<br>0.60	0.60<br>0.38<br>0.27	1.64<br>1.36<br>1.30	0.95<br>0.32<br>0.21
Genotype combination of *BRM*-741 and *BRM*-1321 (0=Others; 1=Both Ins/Ins)	1 vs 0	0.65	0.31	1.26	0.23
Genotype combination of *BRM*-741 and *BRM*-1321 (0=Both Del/Del; 1=Others)	1 vs 0	0.86	0.54	1.40	0.55
Genotype combination of *BRM*-741 and *BRM*-1321<br>(0=Others; 1=At least one Ins/Ins)	1 vs 0	0.69	0.44	1.07	0.10
BRM-741 (dominant model; 0=Del/Del; 1=Others)	1 vs 0	0.69	0.46	1.06	0.09
BRM-741 (recessive model; 0=Others; 1=Ins/Ins)	1 vs 0	0.75	0.45	1.22	0.25
BRM-741 (additive model; 0=Del/Del; 1=Ins/Del; 2=Ins/Ins)	2 vs 1 vs 0	0.78	0.59	1.03	0.08
BRM-1321 (dominant model; 0=Del/Del; 1=Others)	1 vs 0	0.85	0.56	1.28	0.43
BRM-1321 (recessive model; 0=Others; 1=Ins/Ins)	1 vs 0	0.64	0.36	1.07	0.10
BRM-1321 (additive model; 0=Del/Del; 1=Ins/Del; 2=Ins/Ins)	2 vs 1 vs 0	0.82	0.62	1.08	0.15

CI, confidence interval; Del, deletion; Ins, insertion; OR, odds ratio. P values < 0.05 are shown in bold.

[*] Adjusted for age, sex, number of first degree relatives with colorectal cancer, smoking status, and body mass index.
