# Supplementary material for: Two functional indel polymorphisms in the promoter region of the Brahma gene (BRM) and disease risk and progression-free survival in colorectal cancer
Source: PLoS One. 2018 Jun 12;13(6):e0198873. doi: 10.1371/journal.pone.0198873 (PMC5997361; doi:10.1371/journal.pone.0198873)
Supplement: S3 Table — (PDF) [file pone.0198873.s003.pdf]

S3 Table. Results of the multivariate logistic regression analyses for male and female patients.

A. Male cases (n=255) + male controls (n=242)					
			95% CI		
**Variables**	**Category**	*** OR**	**lower**	**higher**	**p value**
BRM-741 (co-dominant model; 0=Del/Del; 1=Ins/Del; 2=Ins/Ins)	1 vs 0	1.00	0.65	1.53	0.99
	2 vs 0	1.07	0.64	1.79	0.79
BRM-1321 (co-dominant model; 0=Del/Del; 1=Ins/Del; 2=Ins/Ins)	1 vs 0	1.14	0.76	1.73	0.52
	2 vs 0	1.10	0.66	1.85	0.71
Genotype combination of *BRM*-741 and *BRM*-1321 (0=Both Del/Del; 1=No Ins/Ins; 2=One Ins/Ins; 3=Both Ins/Ins)	1 vs 0	1.17	0.72	1.91	0.52
	2 vs 0	1.42	0.81	2.52	0.22
	3 vs 0	1.02	0.51	2.05	0.95
Genotype combination of *BRM*-741 and *BRM*-1321 (0=Others; 1=Both Ins/Ins)	1 vs 0	0.86	0.47	1.56	0.62
Genotype combination of *BRM*-741 and *BRM*-1321 (0=Both Del/Del; 1=Others)	1 vs 0	1.21	0.77	1.91	0.41
Genotype combination of *BRM*-741 and *BRM*-1321 (0=Others; 1=At least one Ins/Ins)	1 vs 0	1.14	0.77	1.68	0.51
BRM-741 (dominant model; 0=Del/Del; 1=Others)	1 vs 0	1.02	0.68	1.53	0.92
BRM-741 (recessive model; 0=Others; 1=Ins/Ins)	1 vs 0	1.07	0.70	1.66	0.75
BRM-741 (additive model; 0=Del/Del; 1=Ins/Del; 2=Ins/Ins)	2 vs 1 vs 0	1.03	0.80	1.33	0.80
BRM-1321 (dominant model; 0=Del/Del; 1=Others)	1 vs 0	1.13	0.77	1.67	0.53
BRM-1321 (recessive model; 0=Others; 1=Ins/Ins)	1 vs 0	1.02	0.65	1.60	0.94
BRM-1321 (additive model; 0=Del/Del; 1=Ins/Del; 2=Ins/Ins)	2 vs 1 vs 0	1.06	0.82	1.37	0.65
**B. Female cases (n=172) + female controls (n=166)**					
			**95% CI**		
**Variables**	**Category**	*** OR**	**lower**	**higher**	**p value**
BRM-741 (co-dominant model; 0=Del/Del; 1=Ins/Del; 2=Ins/Ins)	1 vs 0	1.14	0.67	1.97	0.62
	2 vs 0	0.75	0.38	1.46	0.40
BRM-1321 (co-dominant model; 0=Del/Del; 1=Ins/Del; 2=Ins/Ins)	1 vs 0	1.29	0.78	2.16	0.32
	2 vs 0	0.69	0.35	1.34	0.28

Genotype combination of *BRM*-741 and *BRM*-1321 (0=Both Del/Del; 1=No Ins/Ins; 2=One Ins/Ins; 3=Both Ins/Ins)	1 vs 0	1.66	0.87	3.20	0.12
	2 vs 0	1.16	0.54	2.52	0.70
	3 vs 0	0.69	0.26	1.75	0.44
Genotype combination of *BRM*-741 and *BRM*-1321 (0=Others; 1=Both Ins/Ins)	1 vs 0	0.49	0.22	1.06	0.08
Genotype combination of *BRM*-741 and *BRM*-1321 (0=Both Del/Del; 1=Others)	1 vs 0	1.38	0.75	2.59	0.31
Genotype combination of *BRM*-741 and *BRM*-1321 (0=Others; 1=At least one Ins/Ins)	1 vs 0	0.66	0.40	1.09	0.11
BRM-741 (dominant model; 0=Del/Del; 1=Others)	1 vs 0	1.01	0.61	1.70	0.96
BRM-741 (recessive model; 0=Others; 1=Ins/Ins)	1 vs 0	0.69	0.39	1.20	0.19
BRM-741 (additive model; 0=Del/Del; 1=Ins/Del; 2=Ins/Ins)	2 vs 1 vs 0	0.88	0.63	1.22	0.45
BRM-1321 (dominant model; 0=Del/Del; 1=Others)	1 vs 0	1.09	0.67	1.77	0.72
BRM-1321 (recessive model; 0=Others; 1=Ins/Ins)	1 vs 0	0.59	0.33	1.07	0.08
BRM-1321 (additive model; 0=Del/Del; 1=Ins/Del; 2=Ins/Ins)	2 vs 1 vs 0	0.89	0.64	1.22	0.46

CI, confidence interval; Del, deletion; Ins, insertion; OR, odds ratio.

* Adjusted for age, number of first degree relatives with colorectal cancer, smoking status, and body mass index.
