# Supplementary material for: Two functional indel polymorphisms in the promoter region of the Brahma gene (BRM) and disease risk and progression-free survival in colorectal cancer
Source: PLoS One. 2018 Jun 12;13(6):e0198873. doi: 10.1371/journal.pone.0198873 (PMC5997361; doi:10.1371/journal.pone.0198873)
Supplement: S4 Table — (PDF) [file pone.0198873.s004.pdf]

S4 Table. Results of the age-stratified multivariate Cox regression (survival) analyses in the male and female sub-cohorts.

A. Male cases (n=255)						
			95% CI			
**Variables**	**Category**	*** HR**	**lower**	**higher**	**p value**	**p value for PH assumption test**
BRM-741 (co-dominant model; 0=Del/Del; 1=Ins/Del; 2=Ins/Ins)	1 vs 0	0.54	0.34	0.88	**0.01**	0.45
	2 vs 0	1.29	0.77	2.17	0.33	0.70
BRM-741 (dominant model; 0=Del/Del; 1=Others)	1 vs 0	0.73	0.48	1.13	0.16	0.67
BRM-741 (recessive model; 0=Others; 1=Ins/Ins)	1 vs 0	1.84	1.17	2.90	**0.01**	0.89
BRM-741 (additive model; 0=Del/Del; 1=Ins/Del; 2=Ins/Ins)	2 vs 1 vs 0	1.09	0.81	1.47	0.56	0.76
BRM-1321 (co-dominant model; 0=Del/Del; 1=Ins/Del; 2=Ins/Ins)	1 vs 0	0.96	0.61	1.51	0.86	0.05
	2 vs 0	1.01	0.57	1.78	0.97	0.25
BRM-1321 (dominant model; 0=Del/Del; 1=Others)	1 vs 0	0.97	0.64	1.48	0.90	0.05
BRM-1321 (recessive model; 0=Others; 1=Ins/Ins)	1 vs 0	1.03	0.62	1.72	0.90	0.77
BRM-1321 (additive model; 0=Del/Del; 1=Ins/Del; 2=Ins/Ins)	2 vs 1 vs 0	1.00	0.75	1.32	0.99	0.14
Genotype combination of *BRM*-741 and *BRM*-1321 (0=Both Del/Del; 1=No Ins/Ins; 2=One Ins/Ins; 3=Both Ins/Ins)	1 vs 0	0.64	0.38	1.09	0.10	0.99
	2 vs 0	0.94	0.52	1.69	0.84	0.61
	3 vs 0	1.23	0.61	2.51	0.56	0.94
Genotype combination of *BRM*-741 and *BRM*-1321 (0=Others; 1=Both Ins/Ins)	1 vs 0	1.56	0.84	2.88	0.16	0.81
Genotype combination of *BRM*-741 and *BRM*-1321 (0=Both Del/Del; 1=Others)	1 vs 0	0.78	0.49	1.26	0.31	0.78
Genotype combination of *BRM*-741 and *BRM*-1321 (0=Others; 1=At least one Ins/Ins)	1 vs 0	1.37	0.9	2.09	0.14	0.71
**B. Female cases (n=171)**						
			95% CI			
**Variables**	**Category**	*** HR**	**lower**	**higher**	**p value**	**p value for PH**

						**assumption test**
BRM-741 (co-dominant model; 0=Del/Del; 1=Ins/Del; 2=Ins/Ins)	1 vs 0	0.96	0.50	1.84	0.89	0.95
	2 vs 0	0.56	0.22	1.39	0.21	0.13
BRM-741 (dominant model; 0=Del/Del; 1=Others)	1 vs 0	0.83	0.44	1.54	0.55	0.60
BRM-741 (recessive model; 0=Others; 1=Ins/Ins)	1 vs 0	0.57	0.25	1.30	0.18	0.09
BRM-741 (additive model; 0=Del/Del; 1=Ins/Del; 2=Ins/Ins)	2 vs 1 vs 0	0.78	0.52	1.18	0.24	0.23
BRM-1321 (co-dominant model; 0=Del/Del; 1=Ins/Del; 2=Ins/Ins)	1 vs 0	0.83	0.44	1.59	0.58	0.21
	2 vs 0	0.85	0.34	2.11	0.72	1.00
BRM-1321 (dominant model; 0=Del/Del; 1=Others)	1 vs 0	0.84	0.45	1.55	0.57	0.30
BRM-1321 (recessive model; 0=Others; 1=Ins/Ins)	1 vs 0	0.95	0.42	2.16	0.90	0.54
BRM-1321 (additive model; 0=Del/Del; 1=Ins/Del; 2=Ins/Ins)	2 vs 1 vs 0	0.9	0.57	1.41	0.64	0.68
Genotype combination of *BRM*-741 and *BRM*-1321 (0=Both Del/Del; 1=No Ins/Ins; 2=One Ins/Ins; 3=Both Ins/Ins)	1 vs 0	0.65	0.30	1.39	0.27	0.24
	2 vs 0	0.45	0.17	1.18	0.10	0.49
	3 vs 0	0.61	0.16	2.31	0.47	0.94
Genotype combination of *BRM*-741 and *BRM*-1321 (0=Others; 1=Both Ins/Ins)	1 vs 0	0.94	0.29	3.07	0.91	0.61
Genotype combination of *BRM*-741 and *BRM*-1321 (0=Both Del/Del; 1=Others)	1 vs 0	0.59	0.28	1.21	0.15	0.56
Genotype combination of *BRM*-741 and *BRM*-1321 (0=Others; 1=At least one Ins/Ins)	1 vs 0	0.68	0.34	1.35	0.27	0.08

CI, confidence interval; Del, deletion; HR, hazard ratio; Ins, insertion; PH, proportional hazard. P value < 0.05 are shown in bold. P-values are rounded to two decimals.

[*] Age-stratified Cox models adjusted for disease stage, tumor location, microsatellite instability (MSI) status, and treatment with adjuvant chemotherapy status.
