# Supplementary material for: Two functional indel polymorphisms in the promoter region of the Brahma gene (BRM) and disease risk and progression-free survival in colorectal cancer
Source: PLoS One. 2018 Jun 12;13(6):e0198873. doi: 10.1371/journal.pone.0198873 (PMC5997361; doi:10.1371/journal.pone.0198873)
Supplement: S5 Table — (PDF) [file pone.0198873.s005.pdf]

S5 Table. Results of the age-stratified multivariate Cox regression (survival) analyses in the colon and rectal cancer sub-cohorts.

**A. Colon cases (n=280)**						
			**95% CI**			
**Variables**	**Category**	*** HR**	**lower**	**higher**	**p value**	**p value for PH assumption test**
BRM-741 (co-dominant model; 0=Del/Del; 1=Ins/Del; 2=Ins/Ins)	1 vs 0	0.76	0.46	1.26	0.28	0.42
	2 vs 0	0.97	0.53	1.77	0.91	0.43
BRM-741 (dominant model; 0=Del/Del; 1=Others)	1 vs 0	0.81	0.50	1.31	0.39	0.37
BRM-741 (recessive model; 0=Others; 1=Ins/Ins)	1 vs 0	1.17	0.71	1.93	0.54	0.71
BRM-741 (additive model; 0=Del/Del; 1=Ins/Del; 2=Ins/Ins)	2 vs 1 vs 0	0.97	0.71	1.34	0.87	0.42
BRM-1321 (co-dominant model; 0=Del/Del; 1=Ins/Del; 2=Ins/Ins)	1 vs 0	1.00	0.62	1.62	1.00	0.44
	2 vs 0	1.05	0.56	1.97	0.87	0.36
BRM-1321 (dominant model; 0=Del/Del; 1=Others)	1 vs 0	1.01	0.64	1.61	0.96	0.36
BRM-1321 (recessive model; 0=Others; 1=Ins/Ins)	1 vs 0	1.05	0.61	1.82	0.85	0.54
BRM-1321 (additive model; 0=Del/Del; 1=Ins/Del; 2=Ins/Ins)	2 vs 1 vs 0	1.02	0.75	1.40	0.89	0.34
Genotype combination of *BRM*-741 and *BRM*-1321 (0=Both Del/Del; 1=No Ins/Ins; 2=One Ins/Ins; 3=Both Ins/Ins)	1 vs 0	0.63	0.35	1.14	0.13	0.63
	2 vs 0	0.69	0.35	1.35	0.28	0.26
	3 vs 0	0.93	0.40	2.16	0.87	0.61
Genotype combination of *BRM*-741 and *BRM*-1321 (0=Others; 1=Both Ins/Ins)	1 vs 0	1.34	0.67	2.70	0.41	0.47
Genotype combination of *BRM*-741 and *BRM*-1321 (0=Both Del/Del; 1=Others)	1 vs 0	0.67	0.39	1.18	0.16	0.98
Genotype combination of *BRM*-741 and *BRM*-1321 (0=Others; 1=At least one Ins/Ins)	1 vs 0	1.07	0.68	1.68	0.77	0.19
**B. Rectum cases (n=146)**						
			**95% CI**			
**Variables**	**Category**	*** HR**	**lower**	**higher**	**p value**	**p value for PH**

						**assumption test**
BRM-741 (co-dominant model; 0=Del/Del; 1=Ins/Del; 2=Ins/Ins)	1 vs 0	0.59	0.32	1.07	0.08	0.18
	2 vs 0	1.00	0.52	1.93	1.00	0.72
BRM-741 (dominant model; 0=Del/Del; 1=Others)	1 vs 0	0.71	0.42	1.21	0.21	0.28
BRM-741 (recessive model; 0=Others; 1=Ins/Ins)	1 vs 0	1.29	0.71	2.37	0.40	0.84
BRM-741 (additive model; 0=Del/Del; 1=Ins/Del; 2=Ins/Ins)	2 vs 1 vs 0	0.94	0.65	1.34	0.71	0.55
BRM-1321 (co-dominant model; 0=Del/Del; 1=Ins/Del; 2=Ins/Ins)	1 vs 0	0.90	0.51	1.60	0.73	0.90
	2 vs 0	1.02	0.48	2.17	0.95	0.67
BRM-1321 (dominant model; 0=Del/Del; 1=Others)	1 vs 0	0.93	0.55	1.59	0.80	0.95
BRM-1321 (recessive model; 0=Others; 1=Ins/Ins)	2 vs 1 vs 0	1.08	0.54	2.16	0.83	0.61
BRM-1321 (additive model; 0=Del/Del; 1=Ins/Del; 2=Ins/Ins)	1 vs 0	0.99	0.68	1.43	0.95	0.76
Genotype combination of *BRM*-741 and *BRM*-1321 (0=Both Del/Del; 1=No Ins/Ins; 2=One Ins/Ins; 3=Both Ins/Ins)	1 vs 0	0.64	0.34	1.19	0.16	0.89
	2 vs 0	0.78	0.37	1.63	0.50	0.61
	3 vs 0	1.08	0.42	2.83	0.87	0.99
Genotype combination of *BRM*-741 and *BRM*-1321 (0=Others; 1=Both Ins/Ins)	1 vs 0	1.44	0.60	3.46	0.41	0.88
Genotype combination of *BRM*-741 and *BRM*-1321 (0=Both Del/Del; 1=Others)	1 vs 0	0.72	0.41	1.27	0.25	0.78
Genotype combination of *BRM*-741 and *BRM*-1321 (0=Others; 1=At least one Ins/Ins)	1 vs 0	1.14	0.65	1.98	0.66	0.74

CI, confidence interval; Del, deletion; HR, hazard ratio; Ins, insertion; PH, proportional hazard.

[*] Age-stratified Cox models adjusted for disease stage, microsatellite instability (MSI) status, and treatment with adjuvant chemotherapy status. Please note that in rectum cases, the MSI was not included as a covariate because there were only two patients with the microsatellite instability-high (MSI-H) tumor type.
