# Supplementary figures and images for: Two functional indel polymorphisms in the promoter region of the Brahma gene (BRM) and disease risk and progression-free survival in colorectal cancer
Source: PLoS One. 2018 Jun 12;13(6):e0198873. doi: 10.1371/journal.pone.0198873 (PMC5997361; doi:10.1371/journal.pone.0198873)

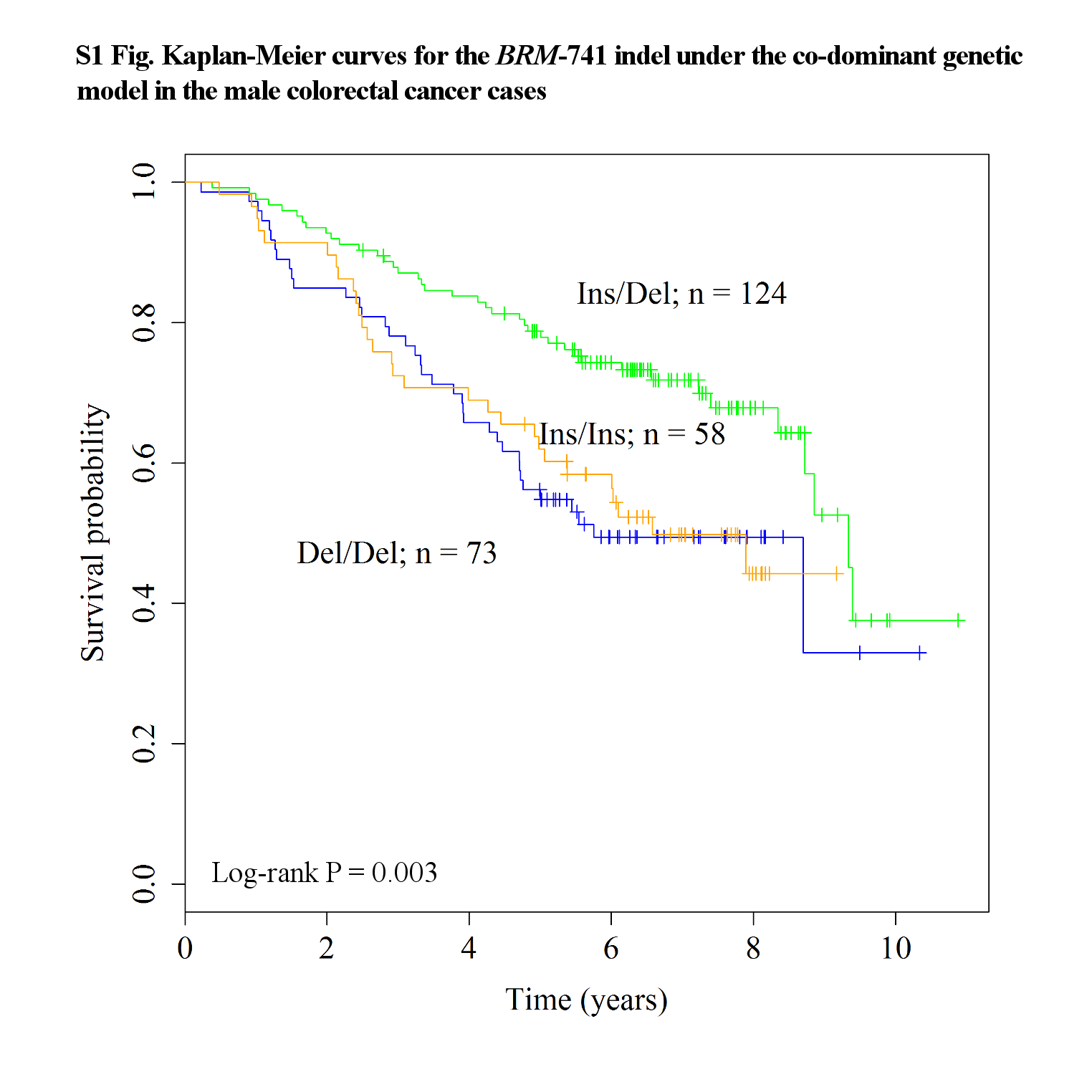

Supplement: S1 Fig — (TIFF) [file pone.0198873.s006.tiff]

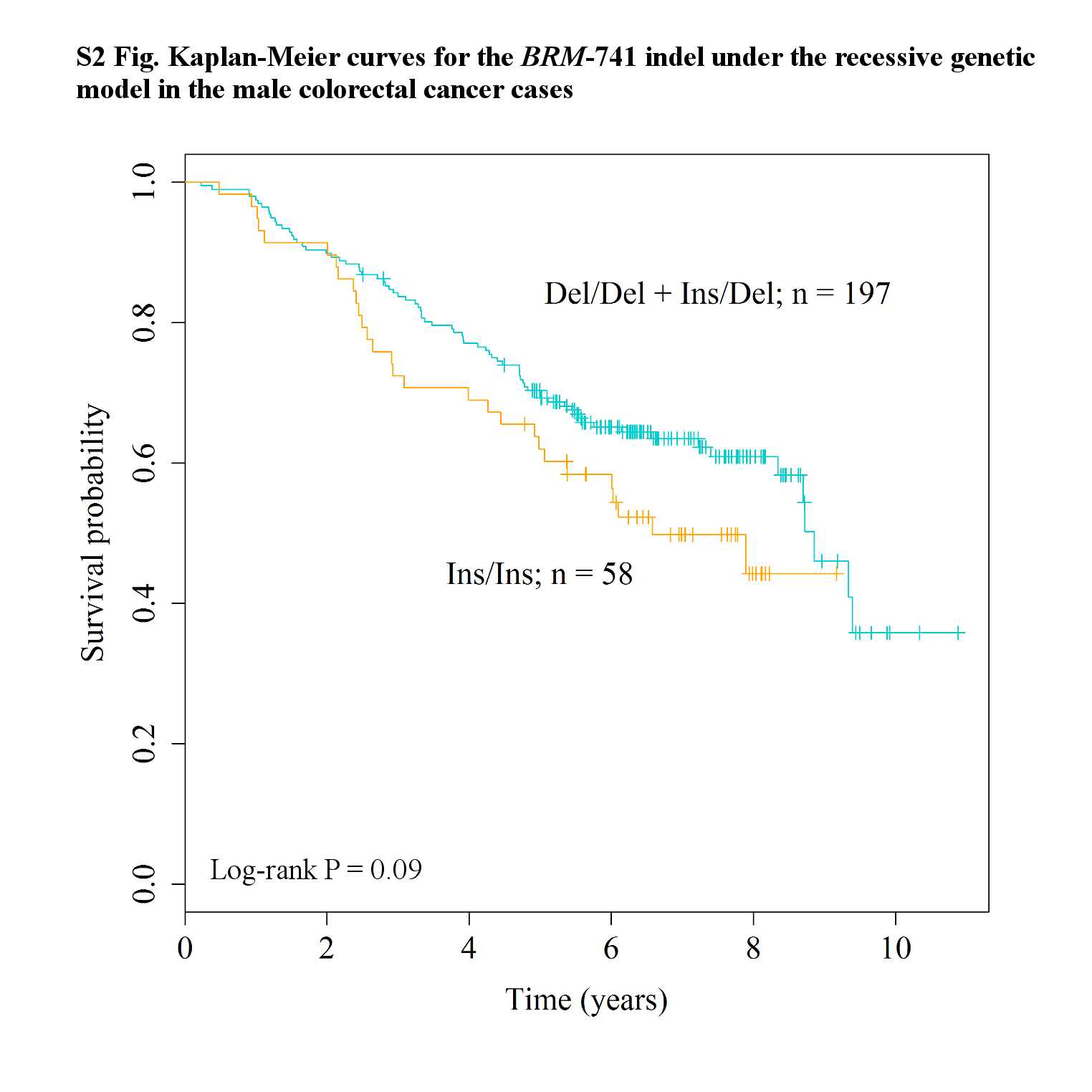

Supplement: S2 Fig — (TIFF) [file pone.0198873.s007.tiff]

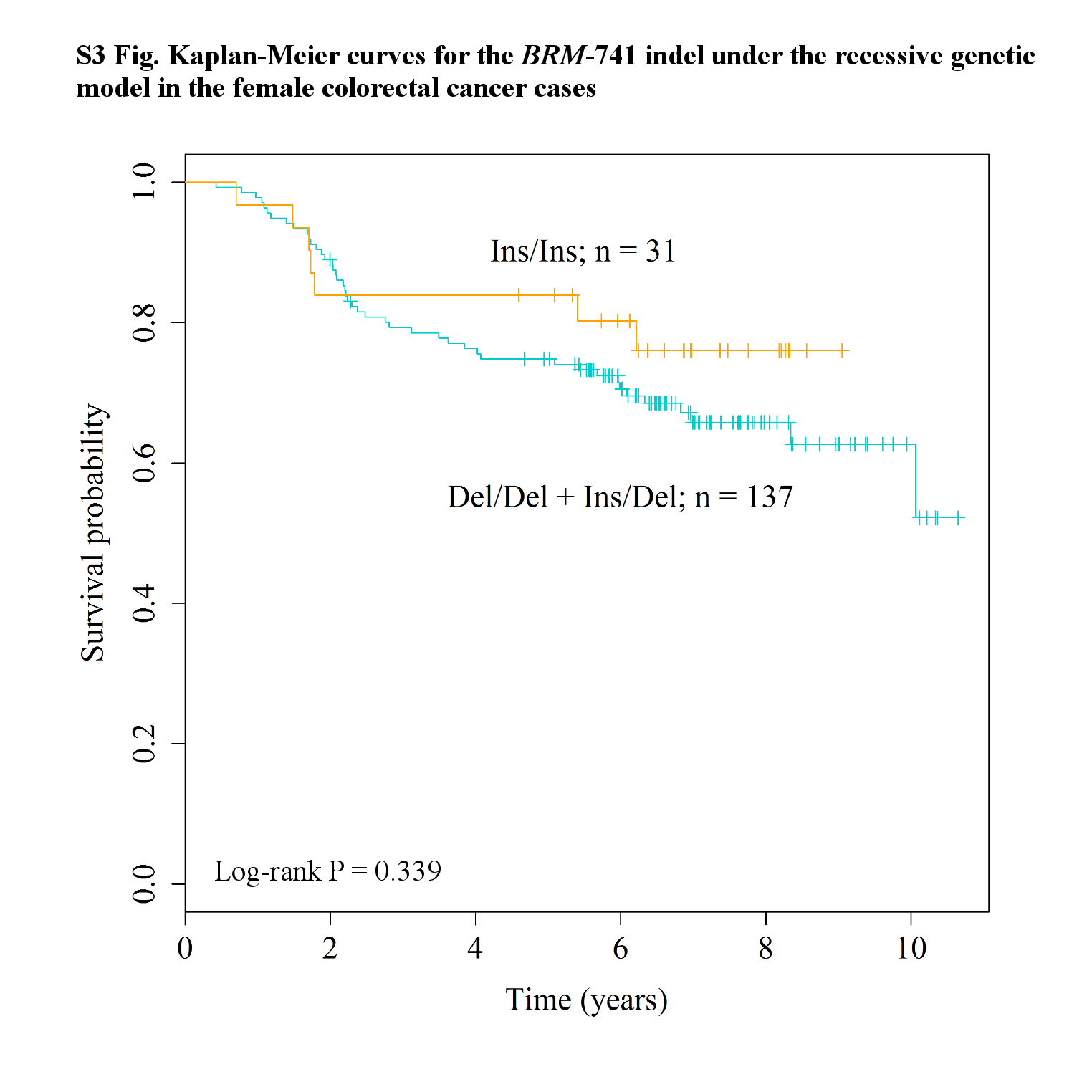

Supplement: S3 Fig — (TIFF) [file pone.0198873.s008.tiff]
